# On the Successful Treatment and Prevention of Consumption and Scrofula; Female Disorders Connected Therewith; Strumous Glandular Swellings, &c.

**Published:** 1844-04

**Authors:** 


Art. XI.—*On the successful Treatment and Prevention of Consumption and Scrofula; Female Disorders connected therewith; Strumous Glandular Swellings, &c.* By J. J. Furnivall, m.d. Second Edition.—*London*, 1844. Small 8vo, pp. 315.

In our Number for April, 1839, we gave an account of this book on its first appearance. As this account was anything but favorable, our readers may wonder how we come to take any further notice of a work which belongs clearly to a class with the writers of which we can have no sympathy,—writers who are either themselves unconsciously deceived through a culpable want of knowledge, or consciously do their best to deceive others by pretending to knowledge which they know they do not possess. To which division of the class Dr. Furnivall belongs, we do not think it worth while to inquire; but we believe the following brief extracts from his new preface will convince all scientific and observant men that we have not classified him wrongly:

"I believe, that if a person apply in time, *deposition of tubercle may be altogether prevented*—that tuberculous deposit, as yet latent, *may be removed by treatment*—and I have notes of cases to prove, that in the second stage, when active congestion or even inflammatory action has taken place around the tuberculous deposit, many a case may, by proper treatment, *be wrested for that time from the grave.*

"The cases which I have attended entitle me to assert, in conclusion, that a proper system of treatment *will be wholly successful, in preventing an attack of consumption, or a recurrence after recovery from a first attack;* and it will be successful, in a great many instances, *where recovery has hitherto been despaired of.*" (Preface, pp. xxii, xxiii, xxiv.)

Our reasons for sacrificing one of our valuable pages for a notice of this poor production, is to exhibit another—and we rather think a novel—aspect of that despicable system of Pseudo-Second-Editions, to which we have before called the attention of our readers. Though Dr. Furnivall's book has all the external characters of a new edition, a new title and titlepage, a new cover, a new preface grandly designated "Prolegomena to Second Edition," and though he boldly tells us that "considerable alterations have been made in the body of the text," the volume before us is, in fact, the identical volume published five years ago, the self-same sheets printed at Hertford in 1838, with merely the alterations already mentioned, and the following very curious changes, to enable the author to keep the word of promise to the ear, but break to the hopes. First we have the "Prolegomena" of six pages, which is new; secondly, we have a reprint of eight pages, (pp. 13-22,) containing a little new matter; thirdly, another half-sheet, (pp. 59-66,) also reprinted; fourthly, we have the reprint of a whole sheet, (pp. 151-168,) also containing a little new matter; and, lastly we have in place of the "conclusion" of the original publication, which is cancelled, five pages substituted, with the enigmatical title of "Summary for Second Edition." Every other page, line, and word in the volume, (which be it remembered extends to 315 pages,) are, as already stated, the identical matter and substance of the old book. And this is what a member of an honorable profession, an "M.D. of the Royal College of Physicians, London," unblushingly calls a Second Edition!

The counterfeit, it must be owned, is on the whole marvellously well executed; and we suspect that few but such cunning old bookwrights as ourselves will detect it: yet, like all shams, there are some incongruities in the fabrication which will reveal its nature to the initiated. Thus, though great pains have evidently been taken to match the new paper with the old, a practised eye will easily discern the difference between them. Again, the old "Table of Contents," retained, doubtless, on an economical principle, true to its original function, still boldly points, in capitals, to p. 318 for "*Conclusion*," when, alas, the *new edition* has its veritable and *boná-fide conclusion* at p. 315! There is no p. 318 except in the deserted storerooms of the Hertford printing office!

